# Multidrug-Resistant *Acinetobacter baumannii*

**DOI:** 10.3201/eid1306.070064

**Published:** 2007-06

**Authors:** Vladimir Krcmery, Erich Kalavsky

**Affiliations:** * St Elizabeth School of Health and Social Sciences, Bratislava, Slovakia; † Trnava University, Trnava, Slovakia

**Keywords:** Acinetobacter baumannii, mortality, multidrug resistance, letter

**To the Editor:** In the January 2007 issue of Emerging Infectious Diseases, Sunenshine et al. (*1*) described their finding of an independent association between patients with multidrug-resistant (MDR) *Acinetobacter* infection and increased hospital and intensive care unit (ICU) length of stay compared with that for patients with antimicrobial drug–susceptible *Acinetobacter* infection. The authors did not, however, find a statistically significant difference in mortality rates between the 2 groups of patients.

*Acinetobacter* infections frequently occur in severely ill ICU patients with other chronic illnesses or prolonged hospitalizations. We analyzed data for 27 neutropenic cancer patients with *A. baumannii*–associated bacteremia (15 with MDR and 12 with drug-susceptible *A. baumannii* infections) but no other chronic illness. We considered *A. baumannii* strains to be MDR if they were resistant to amikacin, meropenem, and ciprofloxacin. Univariate analysis (Epi Info 2000; Centers for Disease Control and Prevention, Atlanta, GA, USA) showed that most of the bacteremic episodes were associated with certain risk factors, such as catheter insertion, neutropenia, acute leukemia, and previous prophylactic treatment with quinolones or therapeutic treatment with cephalosporins or carbapenems (meropenem or imipenem) (Table).

**Table:** Risk factors and outcome for 27 neutropenic cancer patients with bacteremia due to multidrug-resistant (MDR) or drug-susceptible *Acinetobacter baumannii* infection

Characteristic	All patients, no. (%) (N = 27)	Patients with drug-susceptible *A. baumannii,* no. (%)* (n = 12, 44%)	Patients with MDR *A. baumannii*, no. (%)* (n = 15, 56%)
Risk for bacteremia			
Central venous catheter	19 (70.4)	9 (75.0)	10 (66.7)
Acute leukemia	11 (40.7)	6 (50.0)	5 (33.3)
Previous prophylaxis with quinolones	14 (51.9)	8 (66.7)	6 (40.0)
Previous therapeutic treatment with cephalosporins	15 (55.6)	8 (66.7)	7 (46.7)
Previous therapeutic treatment with carbapenems	8 (29.6)	4 (33.3)	4 (26.7)
Outcome			
Septic shock	4 (14.8)	2 (16.7)	2 (13.3)
Death	2 (7.4)	1 (8.3)	1 (6.7)

*Insignificant difference between patients with drug-susceptible infection and those with MDR infection (p<0.05 by univariate analysis).

Septic shock developed in 4 (14.8%) of the 27 neutropenic patients with *A. baumannii*–associated bacteremia, and 2 (7.4%) of the 27 died (Table). However, we did not find a statistically significant association between death among patients with bacteremia caused by MDR *A. baumanni* (1 death) compared with death among those with bacteremia caused by *A. baumannii* strains susceptible to the carbapenems, ciprofloxacin, and amikacin (1 death) (Table). This finding is similar to that described by Sunenshine et al. (*1*) in the general ICU population and in neutropenic cancer patients with bacteremia; however, multivariate analysis was not conducted to control for severity of illness and coexisting illness. In conclusion, neutropenic cancer patients with bacteremia due to MDR *A. baumannii* infection do not appear to be at increased risk for death compared with patients with bacteremia due to antimicrobial drug–susceptible *A. baumannii*.
